# “Sometimes I Get So Extremely Tired”: Sámi Healthcare Staff Experiences of Cultural Load in Practice

**DOI:** 10.1177/23333936241273256

**Published:** 2024-09-17

**Authors:** Tove Mentsen Ness, Grete Mehus

**Affiliations:** 1 Nord University, Levanger, Norway; 2 University of Tromsø, The Arctic University of Norway, Hammerfest, Norway

**Keywords:** culturally safe care, indigenous healthcare staff, burnout, cultural load, Norway

## Abstract

The study aim was to explore how Sámi healthcare staff experience working as an ethnic minority in the Norwegian healthcare system. This was a qualitative focus group and individual interview study inspired by interpretive description, with 14 participants. The results indicate that Sámi healthcare staff experience various degrees of cultural load in their jobs. This was shown through the two themes: “Feeling responsible for Sámi patients and non-Sámi colleagues” and “Feeling exhausted as a Sámi healthcare worker.” To provide culturally safe care for all Sámi patients and their families, it is vital to ensure the well-being of the small number of Sámi healthcare personnel. Therefore, we emphasize the need for training programs for non-Sámi healthcare staff to provide them with the knowledge needed to support their encounters with Sámi patients in culturally safe ways. Sámi healthcare staff cannot take all responsibility for teaching their non-Sámi colleagues and acting as cultural mediators in all situations that non-Sámi staff find challenging. The risk of cultural load and burnout is very present. Nursing departments in universities and leaders in primary and secondary healthcare need to address these issues in order to ensure culturally safe care to all patients and support Sámi healthcare staff.

## Introduction

International studies highlight the fact that Indigenous populations have overall poorer health and social outcomes than the benchmark population (Bartlett et al., 2007), although this is not necessarily the case for the Sámi population (Anderson et al., 2016). However, the standard healthcare services the Indigenous population receives can be perceived as of lower quality (Browne & Fiske, 2001; Tang & Browne, 2008) and can be considered culturally unsafe (Blix & Munkejord, 2022; Ramsden, 2002). A comprehensive census of the Sámi population, the Indigenous population in Norway, Sweden, Finland, and Russia, is not available due to the absence of ethnicity-based registration. Nonetheless, estimates suggest that the Sámi population consists of approximately 50,000 to 65,000 individuals in Norway, 20,000 in Sweden, 8,000 in Finland, and 2,000 in Russia (Vars, 2019), and their homeland, Sápmi, extends across those four countries. The population is not homogeneous, comprising several subgroups with their own language and customs (Dagsvold et al., 2015). Many Sámi have lost their mother tongue due to a harsh colonization process, especially from 1850 onward (Minde, 2003), and many Sámi are now learning their language due to a recent revitalization process.

In Norway, healthcare services are offered to all in need of care, irrespective of their cultural background or where they live (Dagsvold et al., 2015; Health and Care Services Act, 2011; Munkejord et al., 2018; Vabø et al., 2013), and this also applies to the Indigenous people of Norway, the Sámi. Furthermore, the Sámi people in Norway have the right to receive healthcare services adapted to their cultural and linguistic background, as highlighted in the Sámi Act (Sámi Act, 1987), the ILO Convention No. 169 on Indigenous Rights, and the Patient Rights Act, § 3-2 and 3-5 (Patient and User Rights Act, 1999), although this has not yet been consistently implemented. Instead, the same standard healthcare services developed for the majority population are offered to all (Devik & Olsen, 2020; Mehus et al., 2018; Melbøe, 2018; Ness et al., 2015; Nymo, 2011). Qualitative studies indicate that Sámi patients may feel neglected when receiving care (Ness & Munkejord, 2022). This may be because there is no possibility to use their Sámi language in healthcare settings. For example, interpreters are not offered, and there is no access to medical information in Sámi (Engnes et al., 2021). As a result, Sámi patients have reported that they sometimes feel that Norwegian healthcare facilities are neglecting all aspects of being Sámi, which may lead to a culturally unsafe environment (Daerga et al., 2012; Engnes et al., 2021; Mehus et al., 2019; Nystad et al., 2008). Older South Sámi may also feel deprioritized and misunderstood by healthcare professionals, who generally have little or no knowledge of Sámi history and culture. Additionally, they worry that as Sámi they will not be accepted if they 1 day move into a nursing home (Ness & Munkejord, 2022). This could be because of healthcare workers’ lack of competence in Sámi language and culture and their failure to engage in what Sámi patients actually need in healthcare situations (Engnes et al., 2021). Healthcare staff emphasize that this occurs due to a lack of discussion and focus on Sámi culture, art and music in clinical care (Engnes et al., 2021; Hämäläinen et al., 2017, 2020). Mehus et al. (2019) stresses that when staff fail to understand a Sámi patient’s symptoms and experiences, the Sámi patient could interpret this as disrespect or a failure to understand their identity and background, which therefore could imply a culturally unsafe environment.

Studies further indicate that Sámi patients may experience healthcare encounters as culturally unsafe when they cannot use their mother tongue (Engnes et al., 2021; Mehus et al., 2018), while other studies indicate that not all bilingual Sámi feel the need to speak Sámi in healthcare settings (Dagsvold et al., 2015; Ness et al., 2020), since having competent staff is more important than speaking Sámi (Ness et al., 2020). These results highlight that Sámi have different needs regarding the use of their mother tongue and what they find culturally unsafe in healthcare encounters. To ensure that Sámi patients have equitable and culturally safe access to healthcare services, their different cultural and linguistic needs must be addressed (Daerga et al., 2012; Ness et al., 2020).

Indigenous healthcare staff often act as cultural mediators between patients of Indigenous background and non-Indigenous staff, and this work means that they often bear the cultural load in their workplace (Komene et al., 2023). In the Norwegian healthcare system, for example, there is no compensation for the additional expertise possessed by Sámi healthcare staff, or for this added responsibility. Yet Sámi nurses who are called upon and utilized in various hospital wards as interpreters or cultural mediators rarely refuse these requests although they find that this disrupts their work plan for the day, which may make them feel overloaded (Engnes et al., 2021; Jumbunna Institute for Indigenous Education and Research, 2022; Sivertsen et al., 2023). While Indigenous healthcare staff can play an important role in providing culturally safe care, expectations for Indigenous staff to mediate in the care of Indigenous patients has been viewed as a form of racism (Mentzel, 2022; Sivertsen et al., 2023).

The essence of the concept of cultural safety in clinical practice refers to the expectation that healthcare staff treat patients with respect, trust, and social justice, while practicing self-awareness and self-reflection in their encounters with patients (Williams et al., 2021). This is especially related to their power positions as non-indigenous and as privileged in the Western medical system. Although cultural safety and learning outcomes that address Sámi issues have been included in various new curricula for health professional education in Norway since 2019 (Eriksen et al., 2017; Lovdata, 2017, 2019), there are indications that there is still a need for healthcare staff with cultural competence in encounters with Sámi patients (Truth and Reconciliation Commission, 2023).

It is therefore essential to understand how Sámi healthcare staff with this vital cultural competence experience their work situation in the majority healthcare services in Norway. Most healthcare staff in Norway do not have an Indigenous background, so how do Sámi healthcare staff experience working as a minority in the healthcare system? What are their feelings of responsibility for Sámi patients? How do they find working with healthcare staff whose background differs from theirs? To address these questions, the aim of this study is to explore how Sámi healthcare staff experience working as members of an ethnic minority in the Norwegian healthcare system.

## Method

This study was inspired by interpretive description (Thorne, 2016). Interpretive description can be seen as a sensitive approach to qualitative work when there is a need to enhance understanding of what may take place within the field of practice by uniting a specific practice situation with theory. The findings could therefore be seen as specifically sensitive to the unique situations where healthcare staff perform their work (Pesut et al., 2020), as in this study. One of the strengths of interpretive description is that it is based on a small-scale qualitative investigation of a clinical phenomenon, with the aim of capturing themes and patterns within subjective perceptions which can improve clinical understanding in the field of practice (Thorne et al., 2004). Focus groups and individual interviews were conducted to elicit the participants’ experiences (Krueger & Casey, 2015).

### Recruitment

Participants were recruited through the first author’s (TMN) acquaintances in the Sámi community, as she has conducted several studies in Sápmi. They provided names of possible participants. Additionally, the first author (TMN) informed others about the study at different conferences, where healthcare staff, who later became participants, contacted her afterward by telephone or email. Some of them also provided names of possible participants; in this way, a form of snowballing was used in the recruitment process in this study (Streeton et al., 2004). The data were collected during the spring of 2022. Inclusion criteria for taking part in this study were having Sámi background and experience as a healthcare worker in Norwegian healthcare services. These criteria were requested in the recruiting process, and the participants in this study self-defined as Sámi.

### Participants

Fourteen healthcare staff of North, Lule and South Sámi background aged between 32 and 68 years agreed to participate in the study. These 11 women and 3 men had various professional backgrounds, including registered nurses, physiotherapists, and auxiliary nurses/state enrolled nurses. They had broad work experience, ranging from central and local hospitals to long-term primary care, and in various positions. All participants had met Sámi patients, but to various degrees, varying from regularly working with Sámi patients to just meeting them occasionally.

### Individual and Focus Group Interviews

In order to facilitate an environment where participants could discuss and share perceptions and views without pressure to reach a consensus, focus groups (Krueger & Casey, 2015) were chosen. When these are held several times with different participants, it enables the researchers to identify trends and patterns in the perceptions of the participants (Krueger & Casey, 2015). Five focus group interviews with thirteen participants were conducted; the size of each focus group varied from two to four participants. The aim was to conduct only focus group interviews, but due to unexpected circumstances and a lack of time, three individual follow-up interviews were performed afterward.

One focus group interview was conducted via Microsoft Teams, which is a digital workspace. Three focus groups were held face-to-face, and one was a hybrid, where one participant participated digitally via Teams, while the other two participants and the first author met face-to-face. Because the participants lived far from each other and worked shifts, it was not feasible to meet face-to-face. The focus group interviews lasted from 60 to 90 min, while the individual interviews took from 35 to 70 min. All interviews were conducted and transcribed verbatim by the first author (TMN). The participants were asked to discuss how they experienced working as Sámi healthcare staff in the majority Norwegian healthcare system. All participants were also asked to reflect on the same topics in the individual and focus group discussions. Participants were asked, for example, about their collaboration with colleagues and leaders and whether their competence as a Sámi healthcare worker was used.

### Data Analysis

The data were analyzed guided by data analysis strategies suggested for interpretive description as described by Thorne (2016). In the first phase of the analysis process, the authors (TMN, GM) read the transcripts several times, and met digitally on Microsoft teams in order to: (1) become familiar with the data as a whole and (2) generate a shared understanding of the data. Numerous meetings and discussions between the authors led to the identification of key units that related to the aim of this study. These discussions were guided by questions such as “What is going on here?” The next step in the analysis consisted of broad inductive coding of the text units. This was done in order to avoid narrowing the data and interpreting the details too early in the analysis process. In these repeated meetings the authors had numerous discussions to validate codes and to reach agreement on the interpretation of the data. This resulted in overlapping codes being collapsed into broader themes. These themes were discussed several times, and sub-themes and themes were further discussed and organized throughout the whole analysis, always with the research aim in mind. The entire analysis was conducted in a reflective manner, both individually and in digital collaboration. The numerous discussions and continued reflections on the entire data set and previous notes led to a consensus on themes and clearer and more precise names for the themes and sub-themes.

### Ethical Considerations and Roles in the Research Team

The participants in this study were guaranteed anonymity, and no names were included in the interview transcriptions. Informal oral consent was obtained from participants before they joined the study. Most participants came from small rural communities, so in order to protect their anonymity, we had to reassure that them that no situations or descriptions would be recognizable. Therefore, some of the quotes were reconstructed with respect to, for example, gender and place. Both Lee (1998) and Swan and Hobbs (2017) stress that this is a challenge when conducting research in rural and transparent communities. Consequently, general titles and identification numbers, such as participant 1, 2, and 3, were used. The study was assessed by the Norwegian Agency for Shared Services in Education and Research (SIKT) (No. 788125) and conducted in accordance with the Declaration of Helsinki (World Medical Association, 2013). All data were collected and transcribed verbatim by the first author (TMN), but both authors (TMN, GM) contributed to the analysis of the data and the writing and revision of this article. Both authors (TMN, GM) are experienced, privileged, registered nurses and teachers who have worked in rural settings and met Sámi patients. One author [GM] has a mixed ethnic background, and the other [TMN] is Norwegian. Both authors have conducted research in Sápmi, but because neither of them speak Sámi, the first author [TMN] conducted the individual and focus group interviews in Norwegian. The analysis was conducted from an outsider (non-Indigenous) position, and influenced by the researchers’ background and experience, which is described as a privileged and empowered position (Olsen, 2018).

## Results

The analysis resulted in two themes with two associated sub-themes. Please see the Table 1.

**Table 1.** Overview of Themes and Sub-Themes From the Analysis.

Themes	Sub-themes
Feeling responsible for Sámi patients and non-Sámi colleagues	Various ways of facilitating care for present and future Sámi patients
	Feeling responsible for their non-Sámi colleagues
Feeling exhausted as a Sámi healthcare worker	The challenges and contradictions of teaching non-Sámi healthcare staff
	The challenges of working with acquaintances and relatives

### Feeling Responsible for Sámi Patients and Non-Sámi Colleagues

All participants in this study felt various degrees of responsibility to improve healthcare for Sámi patients and their families. However, this work was challenging because requests to facilitate care for Sámi patients were often unstructured and spontaneous, varied considerably, and came from both non-Sámi colleagues and their leaders. As a result, in addition to feeling responsible for Sámi patients, they also felt responsible to support their non-Sámi colleagues in learning how to provide care to present and future Sámi patients.

#### Various Ways of Facilitating Care for Present and Future Sámi Patients

In response to varying types of requests from non-Sámi colleagues and healthcare leaders, participants described ways they took up responsibilities to facilitate better healthcare services for Sámi patients and their families. The participants discussed the need for more involvement from their colleagues and leaders in order to provide more culturally safe healthcare, as stated in educational guidelines and policy documents, and two of them stated:

Participant 1: I think the leaders must be more involved in facilitating safe care for Sámi patients. It must come from the management and down through the system. That is so important.

Participant 2: Yes, I do agree, because then the information will reach everybody, and the non-Sámi staff will understand that this is essential for Sámi patients.

Other participants found that their leader and non-Sámi colleagues requested their expertise as a Sámi healthcare worker if they had a Sámi patient and had a positive view on helping when needed. This was because they often had a better background to understand the Sámi patient and could speak Sámi if the patient preferred that. One participant explained this responsibility in this way:

It’s not that I could provide better care than others [healthcare staff], but that we could say to the patient that I had the same background, and maybe could understand her situation better . . .. yes, and better understand her point of view . . . // . . . so it could be more natural for her to speak to me with the same background as her, because it’s not always easy to talk to anybody about for example alternative treatment, you know.

All participants explained that they wanted to contribute if there was a Sámi patient at their workplace, but some participants found that their knowledge was sometimes overlooked by their leaders. The participants highlighted that their knowledge of Sámi culture was essential to Sámi patients and their families, not only when the leaders or the non-Sámi staff experienced challenging situations, such as when they did not understand when a patient spoke Sámi. However, some participants found that some non-Sámi colleagues had learned a few words of Sámi. One participant explained in this way:

Many Sámi patients only speak their mother tongue if they get dementia, and I’ve found many times that the others [healthcare staff] come running for me and say: “We cannot understand what she’s saying, please help us”. I think that’s awful. But luckily, they’ve learned some Sámi words now, like “I’m thirsty”, “I need to go to the toilet”, and so on.

The participants exhibited compassion and readily stepped in when asked to interpret language and context in instances where their non-Sámi colleagues’ knowledge was insufficient. The participants stated that the need for knowledge of Sámi language and culture may involve a risk for patients, and the various healthcare facilities become highly dependent on the presence of a Sámi healthcare worker, which may not always be possible.

#### Feeling Responsible for Their Non-Sámi Colleagues

All participants in this study expressed feeling responsible for teaching non-Sámi healthcare staff about essential knowledge for encounters with Sámi patients. One participant, who had left her job in healthcare, explained this responsibility in the following way:

I felt as a healthcare worker I had to teach my colleagues about Sámi culture, and I felt that I had a 200% job. First, I had a 100% position in the job I actually had, and then in addition I had to teach my colleagues about Sámi culture as well.

Some participants also stated that there is tacit knowledge that is difficult to learn for non-Sámi staff and they all reported the need for non-Sámi staff to gain more knowledge in order to provide better care to Sámi patients. They therefore felt responsible to try to teach non-Sámi healthcare workers this knowledge. One participant explained it in this way:

It’s like this, there’s tacit knowledge that you just have [as a Sámi healthcare worker], and you work based on that knowledge, and it’s so natural that you don’t think about it, you just do things because you know what to do. But still, I think it’s important to share that expertise so that maybe others [non-Sámi healthcare staff] can also learn, because I think that Sámi healthcare staff can never fulfil the needs of **all** the Sámi patients, we need help or support from non-Sámi staff. So I think it’s important to share one’s expertise with others. So even if they cannot fully understand it [culture], at least they can understand some of it.

The participants were pleased that other Sámi healthcare staff were interested in providing care to Sámi patients, but they also worried about the risk of the care being too person-dependent. Some of them also emphasized that municipalities could have a designated position for a Sámi healthcare worker, which could be used a resource to facilitate care for Sámi patients now and in the future. They then highlighted the need to document how to arrange this to make healthcare services less person- dependent. They thus assumed that unpaid teaching of non-Sámi staff should in principle be managed at the systemic level.

### Feeling Exhausted as a Sámi Healthcare Worker

The additional responsibilities that Sámi healthcare workers had in facilitating culturally safe care for Sámi patients often left them feeling exhausted. They repeatedly encountered non-Sámi healthcare staff in hospitals and home care in and outside Sápmi with limited knowledge of how to relate to Sámi patients. As a result, participants found themselves continually having to explain and educate their colleagues about Sámi culture and traditions, as well as provide cultural translation for patients. Added to this work in healthcare, especially in small rural communities, participants were often called upon to advocate for healthcare for Sámi community members, acquaintances and, at times, for their own relatives. The challenges they encountered in each of these additional areas of work made them feel exhausted and are described in more detail in the following sub-themes.

#### The Challenges and Contradictions of Teaching Non-Sámi Healthcare Staff

Most participants emphasized that they spent considerable time and energy to teach non-Sámi staff how to facilitate care for Sámi patients and to help them appreciate the need for general knowledge about the Sami population and what constitutes culturally appropriate care for this group. This was a challenging task that sometimes exhausted them. The participants realized that it was not possible to teach “everything” about a very different, complex, and diverse culture, despite having the best intentions. This was partly because in all cultures, including Sámi culture, there is tacit, internalized, invisible knowledge that cannot easily be explained. One participant described this dilemma as follows:

Sometimes I get so extremely tired. If you have to walk around all the time and hear your own voice and no matter how hard you try, there are certain things that cannot be transferred. It doesn’t work, you must have grown up in it, you must have it in your spine, you must have it in your blood, it’s not something you can teach to just anyone.

The same participant highlighted that that their non-Sámi colleagues could learn how to treat everybody with respect, and that there are different Sámi cultures and languages, but that some things were not transferable, and this insight sometimes made her feel exhausted. She explained:

You can teach that we speak different Sámi languages, you can teach them that we may have a different kind of culture, but you cannot teach them all these values, all these mindsets. Sometimes I say: I don’t have like a long list of things to explain: How does a Sámi work? It’s just something that’s not transferable. You only have it with you because you were born into this, you are part of something, and we cannot teach that to others. It’s only other Sámi who understand what you’re talking about, and I’ve known that for a very long time and now I’m in a phase where I’m a bit tired of explaining.

The participants discussed the constant demands of being the “Sami voice” at work and in their local community, and talked about putting up with it because they felt a personal obligation to their colleagues and their Sámi patients not to abandon them. One of them said:

I am the Sámi resource in the municipality, and sometimes I get so exhausted. It’s so hard to cope all the time. You get involved in so many personal things, and you have to do that to help and get others [non-Sámi healthcare staff] to understand. And you have to use yourself as an example. I was so tired yesterday, I fell asleep in the chair, you know, because it’s so exhausting, but I have to be the Sámi voice in all settings and. . .//. . . it’s demanding, but I think it’s important, so you must do it. Even if it’s very demanding, you have to do it and be there, because it’s so important [for Sámi patients].

#### The Challenges of Working With Acquaintances and Relatives

Some participants found that working in the healthcare services in the small municipalities where they had grown up could be challenging because they then had to work with their own family members. One participant explained it in this way:

Being professional with your own people, well, we can say you can do that, but I felt that it did something to me. It did something to my feelings, it got into me. There are also different expectations when you work with your own people. They expect you to show up at 3 in the morning or at 7 in the morning. They expect to get an answer and they expect you to assist them in the system. So, you work in a very different way, and you use yourself in a completely different way. I don’t want to go back and work with my own people, it just wears me out too much.

This quote shows that caring for relatives and acquaintances was not a straightforward matter. Several participants explained that this could led to ethically challenging situations. Some participants said that family members wanted them to be their mouthpiece and their Sámi mediator in the healthcare system. In addition to feeling familial obligations to acquaintances and relatives, the participants knew that the non-Sámi healthcare staff did not have the competence they had to provide culturally safe care. It was thus very difficult to set boundaries for themselves when Sámi relatives called on them for help. Not surprisingly, these experiences led to feelings of exhaustion for some participants. One strategy used by some to mitigate this was to work outside their immediate community, such as in another municipality.

## Discussion

The aim of this study was to explore how Sámi healthcare staff experience working as an ethnic minority in the Norwegian healthcare system. The results show that the Sámi healthcare staff experienced various degrees of cultural load in their work, which was shown through the two themes: “*Feeling responsible for Sámi patients and non-Sámi colleagues*” and “*Feeling exhausted as a Sámi healthcare worker*.” Although the participants’ experiences showed individual differences, their experiences of cultural load can be seen through the three levels of healthcare delivery, the overall systemic level, the institutional level, and the individual level. Therefore, although their experiences were on the individual level, the possible solution to their feeling of cultural load may be found on another level. The discussion section is therefore organized in three levels; systemic, institutional, and individual, according to how the findings suggest countermeasures at the various levels.

The systemic level represents the overall health policies, legislation, and guidelines, which provide direction for the delivery of healthcare services and emphasize that all patients have the right to receive healthcare adapted to their cultural background as citizens of Norway (Health and Care Services Act, 2011; Lovdata, 2017; Ministry of Local Government and Modernisation, 2020). The participants in this study found that it could depend on the individual healthcare worker whether Sámi patients received culturally safe care. This was because of the variation in the extent to which the staff used their cultural competence in providing care to Sámi patients. Further, the participants felt that their expertise was rarely used to develop guidelines for tailoring care for Sámi patients. Instead, they were called upon to contribute to care for Sámi patients on a case-by-case basis. Not surprisingly, this led to feelings of exhaustion. Browne et al. (2016) underline that there are several key elements needed to support the health and well-being of Indigenous people, one of which is culturally safe care. In order to provide this, Browne et al. (2016) point out 10 strategies, where the most important factors are tailored care, programs to actively counter racism and discrimination, services in local contexts, and insight into Indigenous cultures and knowledge systems. In other words, health policies, legislation, and guidelines need to be implemented according to the local context. In Norway, this will also require the hiring of Sámi healthcare staff who can act as cultural mediators and interpreters and support the implementation of culturally safe care. Given these additional expectations, providing adequate compensation for this work and recognition in other ways will be important in recruitment and retainment of these healthcare workers.

At the institutional level, the focus is on policies, values, and practices in a healthcare service that shape the perspective of leaders and the institution as a whole. The theme “Feeling responsible for Sámi patients and non-Sámi colleagues” showed that the participants in this study accepted responsibility for various aspects of planning and facilitating care for both present and future Sámi patients, a role that, unlike other health providers, was an additional expectation of their leaders and the institution. While these leaders recognized the need for culturally appropriate care for Sámi patients, they relied on Sámi healthcare staff to provide this care in addition to their normal duties.

Nevertheless, Taylor et al. (2020), based on their study in Australia, emphasize that it can be difficult to recruit and retain healthcare staff with an Indigenous background. Their strategy for achieving positive patient outcomes and a strong Indigenous healthcare workforce is to (a) have a decisive and goal-oriented leadership, (b) commit to an inclusive and enabling culture, (c) facilitate two-way learning, and (d) develop specific support structures appropriate for Indigenous healthcare workers. This could also be done in Norway to comply fully with health policy and legislation as well as in other settings providing care to Indigenous people as recommended by others (Deroy & Schütze, 2019).

Observations of Sami patients in a culturally unsafe environment were stressful for participants and reinforced their view that all healthcare personnel, in general, should have the opportunity to gain knowledge about Sámi culture and language, and how to facilitate care for Sámi patients. Participants pointed to the lack of plans and guidelines for the implementation of the rights of Sámi patients and how to fulfill these rights at various workplaces. This was mentioned even though the interviewer did not ask about it. Browne et al. (2016) suggest that approaches to culturally safe care should be grounded in local Indigenous contexts, in partnership with Indigenous people. Nevertheless, participants cautioned that their Sámi healthcare workers’ knowledge must be used, but not misused. They recommended that Sámi staff be used as a resource, especially to support advanced preparation and planning for culturally safe care and to avoid challenging situations in the care of Sámi patients. The participants wanted leaders in primary and secondary care to collaborate with Sami healthcare staff to support culturally safe care for all patients, including Sámi patients.

At the individual level, the results from this study indicate that when individuals are asked to be cultural mediators at work, employers and colleagues need to be aware of the risk of excessive cultural load (Sivertsen et al., 2023). Sivertsen et al. (2023) emphasize that everyone needs to be a cultural ally and advocate for change, rather than expecting Indigenous healthcare staff, to advocate for change on their own. The study findings also point out the unique challenges and vulnerabilities of Indigenous healthcare staff who live and work in rural areas, where they have additional commitments to ensure access to culturally safe care for people in their community, including relatives. Everybody in small communities knows who works in healthcare. For a rural healthcare worker, this can be perceived as both an advantage and a disadvantage (Bushy, 2008; Scharff, 2010). The benefits and drawbacks may be amplified for rurally located Indigenous healthcare workers.

The risk for burnout among Sami healthcare workers was evident in this study as they attempted to meet requests for assistance from non-Sámi colleagues to help in challenging encounters with Sámi patients when the colleagues. If only Sámi healthcare staff provide care to Sámi patients, non-Sámi staff will continue to have difficulty learning from and reflecting on encounters with Sámi patients. Furthermore, whether Sámi patients receive appropriate care, feel safe, and experience well-being will therefore be arbitrary (Hughes & Farrow, 2006; Johnstone & Kanitsaki, 2006, 2007; Mehus et al., 2019). Together these observations reinforce the need for non-Sámi staff to acquire the necessary knowledge and training to provide culturally safe care. This means that healthcare leaders should include culturally safe care in in-house training sessions. This could also be implemented, for example, in the induction program for new employees as suggested by Heatta et al. (2019).

In other words, there will never be enough Sámi healthcare staff to take care of all Sámi patients in primary and secondary settings and provide them with culturally safe care. For this reason, it would be beneficial to include a strong focus on Sámi patients’ rights and needs in the curricula of bachelor’s and master’s programs in medicine, nursing, and social work (Eriksen et al., 2017; Lovdata, 2017, 2019, 2020; Mehus et al., 2023).

Mentzel (2022) and Sivertsen et al. (2023) suggest that expecting Indigenous healthcare staff to be constantly available for Indigenous patients could be considered racism. In contrast to Mentzel (2022), the participants in this study reported that they appreciated being a “resource” and being called upon when Sámi patients needed healthcare, but sometimes found this exhausting. The balance between using and misusing Sámi healthcare staff is fragile and can lead to burnout, as described in other studies on Indigenous healthcare staff (Conway et al., 2017; Deroy & Schütze, 2019). Strengthening health professional programs is critical to ensuring that Sámi patients receive culturally safe care and that the contributions of Sámi healthcare workers are utilized appropriately and well supported by healthcare teams. This means that it should be opportunities for staff to reflect on their power positions as healthcare workers representing the majority population in an area, and the dominance of the Western medical perspective, which may differ from that of the Sámi lifeworld (Javo, 2020; Williams et al., 2021).

The concept of “two-eyed seeing,” that is, seeing through the lenses of both Indigenous and Western worldviews, should also be introduced to enable healthcare staff to have a better appreciation of the challenges that Sámi healthcare staff experience in working in the Western (Norwegian) healthcare system, but also representing the Sámi culture, and that they use of both perspectives in tailoring care for Sami patients. It is therefore essential that non-Indigenous healthcare workers learn about the assimilation and repression of Sámi culture and language over decades. Against this background, in order to achieve equality in Norwegian healthcare, the staff must no longer relate to all patients in the same way, which could be seen as a subtle ongoing colonization process toward Sámi patients.

The participants wanted to give advice and teach their non-Sámi colleagues how to improve the situation for Sámi patients, but felt that it might be difficult to explain how Sámi culture operates. Speaking on behalf of an ethnic group may therefore present challenges regarding legitimacy, whether conditions are generalizable, and whether one is representing the culture accurately based solely on personal perception. Therefore, knowledge about cultural safety and Sámi patients’ rights must be increased by including it in new regulations common to all healthcare education (Mehus et al., 2023).

## Methodological Considerations

This study aimed to explore how Sámi healthcare staff experience working as an ethnic minority in the Norwegian healthcare system. We cannot conclude that the findings in this study apply to all Sámi healthcare staff in Norway, and the study is therefore not generalizable. While the findings in this study may not capture the experiences of all Sámi healthcare staff, the study provides important insights into the complex situation of working as minority Sámi healthcare staff in the majority Norwegian healthcare system. Both individual and focus group interviews were conducted, which could be seen as a disadvantage. However, this approach to data collection enabled participation of Sámi healthcare workers from across the region. Additionally, Lambert and Loiselle (2008) argue that combining individual interviews and focus groups may also enhance data richness through three main contributions: a productive iterative process, identification of individual and contextual circumstances about a phenomenon and lastly, convergence of a phenomenon, which may enhance the trustworthiness of the findings, which we hope is the result of this study.

## Conclusion and Implications for Practice

The aim of this study was to explore how Sámi healthcare staff experience working as a cultural minority in the Norwegian healthcare system. The results show that the Sámi healthcare staff experienced various degrees of cultural load in their job, which was indicated through the two themes: “Feeling responsible for Sámi patients and their non-Sámi colleagues” and “Feeling exhausted as a Sámi healthcare worker.” Therefore, we emphasize the need for training programs for non-Sámi healthcare staff to acquire the knowledge needed to support their encounters with Sámi patients in culturally safe ways. Sámi healthcare staff cannot take all responsibility for teaching their non-Sámi colleagues and acting as cultural mediators in all situations that non-Sámi staff find challenging. The risk of cultural load and burnout is very present. Nursing departments in universities and leaders in primary and secondary healthcare need to address these issues in order to ensure culturally safe care to all patients and support Sámi healthcare staff.
